# Human-induced pluripotent stem cells-derived retinal pigmented epithelium, a new horizon for cells-based therapies for age-related macular degeneration

**DOI:** 10.1186/s13287-022-02894-0

**Published:** 2022-05-26

**Authors:** Samaneh Dehghan, Reza Mirshahi, Alireza Shoae-Hassani, Masood Naseripour

**Affiliations:** 1 Stem Cell and Regenerative Medicine Research Center, Iran University of Medical Sciences, Tehran, Iran; 2 Eye Research Center, The Five Senses Health Institute, Rassoul Akram Hospital, Iran University of Medical Sciences, Tehran, Iran

**Keywords:** Cell therapy, Age-related macular degeneration, Retinal pigmented epithelium, Induced pluripotent stem cells, Retina, Clinical trial, RPE transplantation, Small chemical molecules

## Abstract

Retinal pigment epithelium (RPE) degeneration is the hallmark of age-related macular degeneration (AMD). AMD, as one of the most common causes of irreversible visual impairment worldwide, remains in need of an appropriate approach to restore retinal function. Wet AMD, which is characterized by neovascular formation, can be stabilized by currently available therapies, including laser photocoagulation, photodynamic therapy, and intraocular injections of anti-VEFG (anti-vascular endothelial growth factor) therapy or a combination of these modalities. Unlike wet AMD, there is no effective therapy for progressive dry (non-neovascular) AMD. However, stem cell-based therapies, a part of regenerative medicine, have shown promising results for retinal degenerative diseases such as AMD. The goal of RPE cell therapy is to return the normal structure and function of the retina by re-establishing its interaction with photoreceptors, which is essential to vision. Considering the limited source of naturally occurring RPE cells, recent progress in stem cell research has allowed the generation of RPE cells from human pluripotent cells, both embryonic stem cells (ESCs) and induced pluripotent stem cells (iPSC). Since iPSCs face neither ethical arguments nor significant immunological considerations when compared to ESCs, they open a new horizon for cell therapy of AMD. The current study aims to discuss AMD, review the protocols for making human iPSCs-derived RPEs, and summarize recent developments in the field of iPSC-derived RPEs cell therapy.
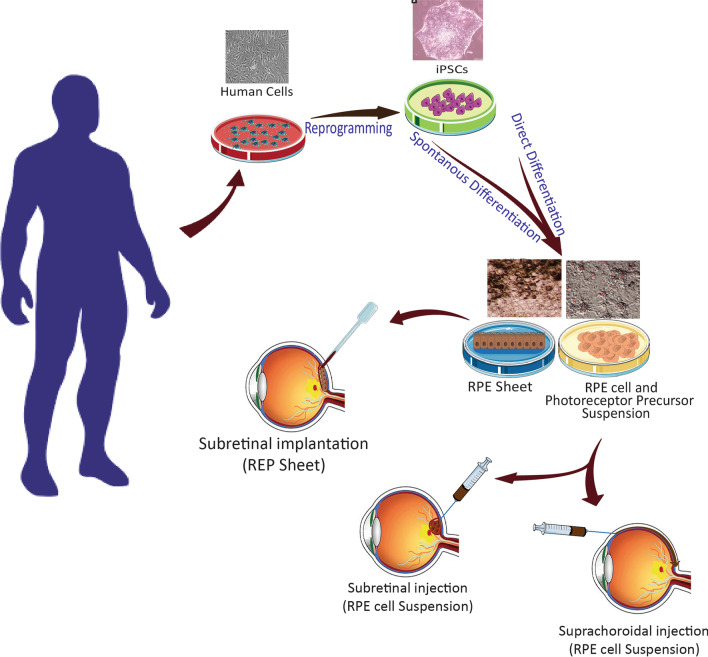

## Introduction

Age-related macular degeneration (AMD), the main cause of central vision loss in patients older than 55Y worldwide, is initiated by the degeneration and loss of the retinal pigmented epithelium (RPE) in the macula caused by diverse mechanisms that remain under investigation [1, 2]. AMD is presented in two forms, neovascular (wet) and non-neovascular (dry). Existing therapies for wet AMD, including intravitreal injection of anti-VEGF (anti-vascular endothelial growth factor), photocoagulation, or both, show only limited effects in terms of both functional and anatomical improvement and just tend to stabilize the disease. On the other hand, dry AMD does not respond to current methods of therapy, and currently, no effective treatments can reverse it, although neuroprotective agents, visual cycle modulators [3], and drugs targeting the complement pathway are under investigation [4]. For many years, visual impairment due to retinal degeneration has been an incredible challenge for ophthalmologists and visual scientists who hope to restore this precious sense [5]. Over the past decade, tissue replacement approaches have given rise to the treatment of immedicable retinal diseases [6]. Stem cells, a nonspecialized immature cells without complex structures, have limitless self-renewal ability and are characterized by the power to differentiate into numerous types of cells in the body [7]. According to “Epigenetic Landscape” by Conrad Waddington [8], in 2006, Yamanaka’s team revolutionized the stem cell field by figuring out that somatic cells can be reprogrammed into embryonic stem cell (ESC)-like cells, called induced pluripotent stem cells (iPSCs) [9]. The possibility to produce patient-specific iPSCs provided a new horizon for both physicians and patients. Since iPSCs bypass many issues and ethics compared to ESCs [10], they show great promise as the main source for cutting-edge cell replacement therapy for different degenerative diseases, including AMD [11].

Technologies have made clinical-grade cell replacement therapies from pluripotent stem cells (both ESCs and iPSCs) possible for AMD. Stem cells can differentiate into bonafide-like RPE cells in vitro, albeit the derivation of RPE from iPSCs is a much faster and more cost-effective approach [1, 12]. One paradigm of pluripotent stem cells differentiation toward RPE cells is “spontaneous” differentiation. However, it is extremely inefficient (1%) and slow in culture [13]. Scientists have been working on the speed and efficacy of RPE production to promote this technology toward clinical practice more quickly and efficiently. Thus, our aims in the present review are to provide a brief overview of AMD, the developed protocols for the differentiation of iPSCs toward RPEs, and summarizing current advancements in the field of iPSC-RPEs transplantation.

## Age-related macular degeneration (AMD)

Age-related macular degeneration (AMD) is the primary cause of permanent central visual loss globally. Clear central vision is needed for daily activities such as driving and reading. “Age-related” means that it occurs in older persons and “macular” comes from “macula,” which is responsible for sharp and high-accuracy vision in the central portion of the visual field [14]. As long as peripheral vision is preserved, AMD does not cause total blindness in patients. Studies confirm the robust relationship between age and AMD, probably as a result of the complicated interaction of genetics, metabolic, and inflammatory mechanisms as well as several environmental factors, including smoking, lifestyle, and nutritional disorders [15].

As the world’s population ages, the incidence rate of AMD will increase significantly, and it has been estimated that it will affect around 288 million people by 2040. AMD is most prevalent in white patients, followed by Asians and Hispanics, and is lowest in the black ethnic group [16]. Visual impairment from advanced AMD is associated with a significant loss of functions, depression, and reduced quality of life [14]. Future socioeconomic and medical challenges associated with AMD will be similar to those of acquired immunodeficiency syndrome, kidney failure, and stroke [17]. Patients may suffer from wet, dry, or both forms of AMD. Advanced stages of AMD manifest as geographic atrophy or neovascular formation. Two categories of dry AMD, “early dry” and “late dry” AMD, are characterized by the formation of drusen and geographic atrophy, respectively. Dry AMD accounts for almost 80% of these patients and is associated with the slow deterioration of the RPE and photoreceptors [4]. Most severe visual loss from this type of AMD is caused by the late stages of dry AMD [18]. Wet AMD is more aggressive associated with sudden worsening of vision which accounts for 20% of this type of degeneration [19]. Clinical diagnosis, based on characteristic findings from dilated retinal examinations, are comprised of extensive small (less than 63 μm), medium (around 63–124 μm), or large drusen (more than 124 μm) [7], geographic atrophy, choroidal neovascularization, or disciform scar formation [20].

## Physiopathology of AMD

AMD physiopathology is not yet completely understood. Research has focused on a variety of mechanisms, including oxidative stress, chronic inflammation, complement cascade, single-nucleotide polymorphism in the complement factor H (*CHF*) gene, and mitochondrial dysfunction [21]. In some patients with wet AMD, an increased level of VEGF causes changes to Bruch’s membrane, which sequentially results in subretinal fragile neovascularization and exudation. They also may be associated with hemorrhage beneath the retina, leading to detachment of the sensory retina, RPE, and subsequent central visual loss. In dry AMD, accumulation of cell debris, called drusen, between the choroid and the retina adversely affects the overlying retina [18]. Oxidative stress is the main contributor to AMD due to high oxygen consumption by the retina [22]. Simply, light-oxidative injury happens once the light interacts with the visual pigments and ultimately leads to the aggregation of the lipofuscin and extracellular drusen formation [23].

## Etiology of AMD

The etiology of AMD may be attributed to genetic-related influences [24]. Several studies have considered the role of genetic variants during the development and progression of AMD, such as complement factor H gene (*CHF*), age-related maculopathy susceptibility gene2 (*ARMS2*), and tissue inhibitor of metalloproteinase 3 (TIMP3) [14]. The most important genetic abnormalities linked with AMD arise in the complement *CHF*, which inhibits the inflammatory cascade that regulates inflammation [4, 14]. The ARMS2 protein that localizes to the mitochondria and contributes to the metabolism of energy is a powerful predictor of AMD, although the precise function of this protein has not yet been discovered [14]. Also, a rare variant of *TIMP3* is strongly related to AMD development [25]. It has been suggested that *TIMP3* modulates not only the action of MMPs but also other molecules, such as VEGF, EGF (epidermal growth factor), and TNF (tumor necrosis factor), and thus, it has a fundamental role in maintaining the homeostasis of RPE extracellular matrix and RPE metabolism in AMD progression [25].

## Function of RPE

**Fig. 1:**
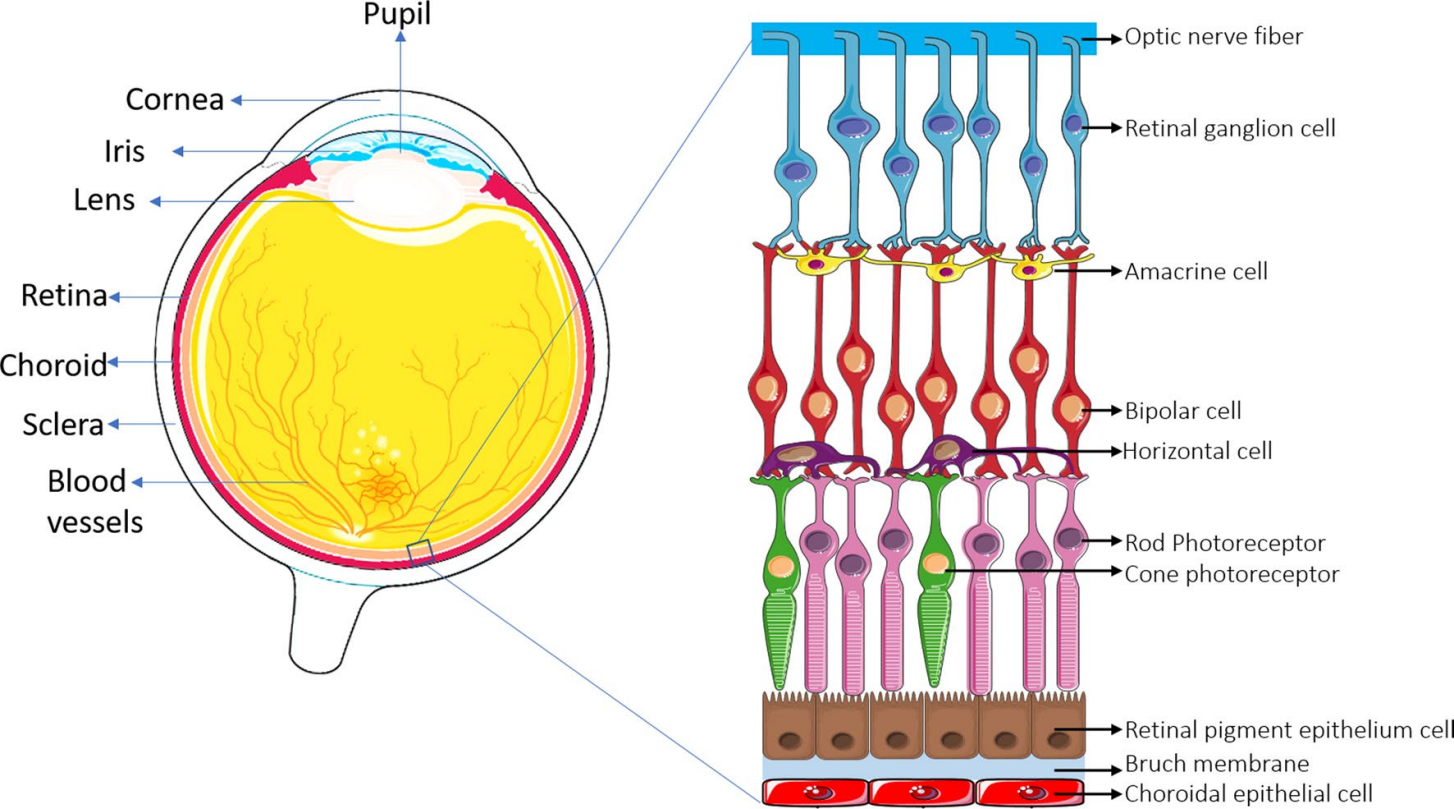
Schematic representation of retinal layers

The pathology hallmark of AMD is RPE–Bruch’s membrane complex damage. The RPE is a post-mitotic single sheet of cells [26] lying at the border between the choriocapillaris and the sensory retina, where the outer blood–retinal barrier (BRB) forms (Fig. 1). The RPE layer is responsible for the immune-privileged state of the eye by releasing immunosuppressive agents [27]. The most important functions of the RPE layer are the regulation of ions, nutrients, water, and waste products transportation to the choroidal vasculature through the Bruch’s membrane, phagocytosis of the outer segment of the photoreceptor (essential for photoreceptor renewal, high-energy light absorption, and protection against light-oxidative damages), re-isomerization of all-*trans*-retinal into 11-*cis*-retinal, and finally, keeping the integrity of the RPE-retina structure by directional secretion of its necessary factors [26]. With age, the permeability of the Bruch’s membrane structure changes and leads to the accumulation of N-retinylidene-N-retinylethanolamine (A2E) and lipofuscin, which are deposited between the Bruch’s membrane and RPE, leading to the formation of yellow drusen. The accumulation of drusen between the RPE and Bruch’s membrane inhibits metabolite transportation to the choroidal vessels and initiates inflammatory cascades. It is also highly phototoxic and has been linked to several oxidative changes, which, in turn, lead to damage or death of RPE and photoreceptors and further geographic atrophy and dysfunction of the Bruch’s membrane [28].

## Current management of AMD

As discussed previously, there are two main categories for AMD: dry or non-neovascular AMD and wet or neovascular AMD. Currently, no effective treatment is available for dry AMD [19]. Although multiple targets such as complement inhibition, neuroprotection, and anti-inflammatory factors have been investigated for treatment of AMD, none have yielded positive results. These treatment failures can be justified by the concept of “the point of no return” in the disease cascade process which has led to irreversible cell loss (i.e., RPE and photoreceptors). The current clinical approach in the management of dry AMD is focused on dietary supplementation to prevent conversion to late stages of the disease without obvious visual benefit [29].

Available therapeutic options for wet AMD focus on limiting the neovascular membrane but do not repair the damage that may have already occurred. First-line therapy for patients who suffer from wet AMD is intravitreal VEGF inhibitors (e.g., ranibizumab, brolucizumab, bevacizumab, and aflibercept) [14].

Photodynamic therapy with/without anti-VEGF medications is another option for the treatment of patients where initial treatment with anti-VEGF was not effective. Thermal laser photocoagulation can result in enlarging scotoma or a new scotoma development, so it is rarely recommended nowadays [30].

## Cell-based therapies for AMD

Cell therapy offers an unlimited source of cells for cell transplantation studies [31]. Currently, retinal cell transplantation, which is differentiated from various stem cells, is a hopeful therapeutic method in ophthalmology [31]. Several different cell types are presently under investigation for clinical cell therapy in AMD. Among all retinal cells, the most common target for cell therapy of AMD studies is the RPE cell [32]. One way of replenishing RPE cells in AMD involves delivering RPE cells to the subretinal space to restore physiological function to the tissue or organ. Retinal progenitor cell (RPC) and RPE produced from ESCs and iPSCs have been suggested as cell sources in preclinical and clinical trials [2, 24]. Stem cells are unspecialized cells of the human body. In addition to having the ability to differentiate into any cell of an organism, they can also self-renew. Pluripotent stem cells (PSCs) can form all germ layers but not extraembryonic structures, such as the placenta. Pluripotent stem cells include embryonic stem cells and induced pluripotent stem cells. Reprogramming of adult cells results in the production of induced pluripotent stem cells (iPSCs) [33, 34].

The use of the ESCs technique has been associated with ethical limitations and immunological complications upon allogeneic transplantation [35] when the origin of the donor cells is not from the recipient patient [36]. iPSCs technology by overcoming to ESC’s ethical challenges has been hailed as an effective replacement for ESCs and a prime candidate cell source for regenerative medicine aims. This technology opens new horizons for scientists in the area of regenerative medicine and cell therapy and provides encouraging results to replace damaged tissues in different pathologic processes [36].

iPSCs are induced reprogramming of differentiated somatic cells back into an embryonic-like pluripotent status. iPSCs technology was established by Shinya Yamanaka, who showed that ectopic expression of four pluripotency transcription factors, termed KLF4, c-MYC, OCT4 and, SOX2, could convert somatic cells to the pluripotent state, which can then be re-differentiated into various desired types of cells [37]. While iPSCs do not exist naturally, any healthy person or patient’s cells can be transformed into iPSCs in a healthy/patient-matched manner. iPSCs could provide an unlimited pool of autologous cells that can be used for transplants without the risk of immune rejection [19]. Easily accessible tissues, such as skin, blood and even urine can be used as a source of adult somatic cells for iPSCs derivation [38].

Retina has a complex architecture made up of the interconnection of a wide variety of cells [39]. Degenerative mechanisms that disrupt this interconnectivity can cause serious visual impairment in patients [40]; thus, future optimizing strategies that potentiate regeneration of the retina are necessary to prevent increases in the burden of retinal diseases [40]. Studies have demonstrated the low clinical efficiency of autologous RPE harvested from healthy locations of the patient’s retina [12, 41]. So, in recent studies, the potential of pluripotent stem cells is being explored for cell therapies in retinal diseases [40].

## iPSCs differentiation to RPE

The most important challenge facing cell therapists in treating AMD is choosing the source of cells and methods to generate bonafide RPE cells. However, since both hESCs and hiPSCs can differentiate toward RPE cells, controlling the potency of hPSCs differentiation into desired cells is one of the important goals of many research teams [13]. Although protocols to generate hiPSC-derived RPE has improved the efficiency of induced RPE since it was first reported in 2004, they are still insufficient and time-consuming. In addition, the iPSC-derived RPE survival rate is limited after in vivo transplantation [42]. Therefore, different laboratories have been working to optimize an efficient and rapid protocol to generate a large-scale RPE cell to shift iPSC-derived RPE toward clinical use. Several initiated studies distinguished straightforward differentiation of RPE from iPSCs. hiPSCs can differentiate toward pigmented RPE either spontaneously or directly [43]. An easy, spontaneous protocol to differentiate PSC (e.g., ESC and iPSC) toward RPE has been reported by a research team, albeit with low efficiency (less than 10%) [28]. In that report, the medium was merely changed to a medium deprived of fibroblast growth factor-2 (FGF-2) with a minor difference in the presented timeline. For preclinical and clinical investigation, it is necessary to expand the RPE cells to obtain a pure RPE cell culture [43]. The “spontaneous” procedure is very slow, operator-dependent, and does not allow for the manufacturing of a sufficient scale cell. To overcome these barriers, researchers have differentiated iPSCs into RPE directly by adding chemical molecules affecting signaling pathways that are recognized to be critical in the development and specification of RPE [44].

**Fig. 2:**
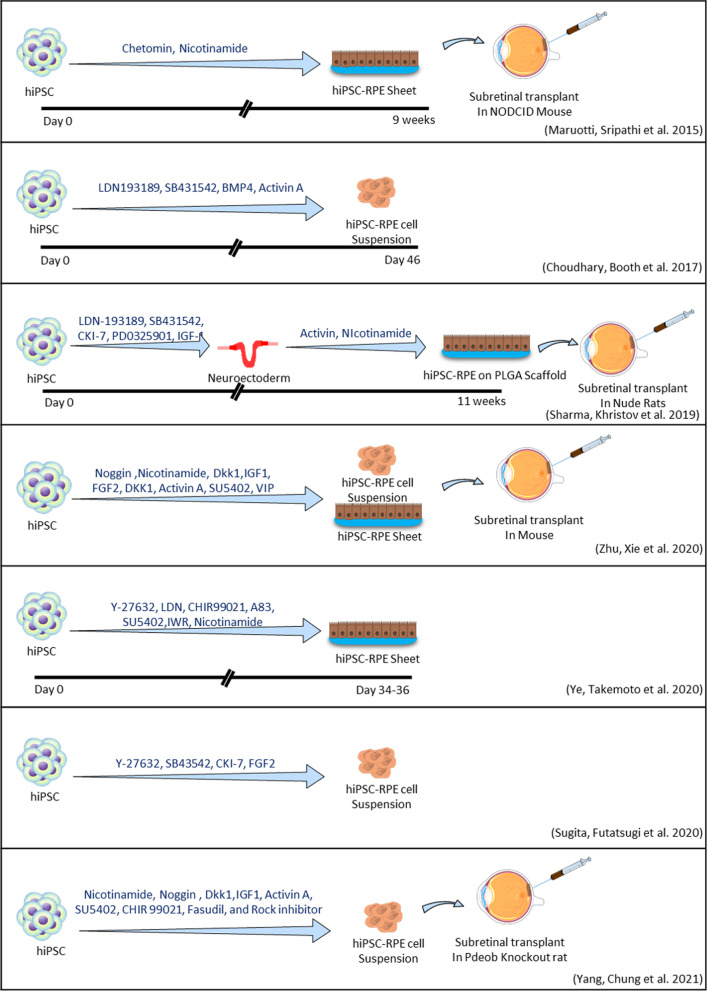
Summary of selected protocols for hiPSC-derived RPE transplant studies using chemical molecules

Various growth factors and chemical molecules have been tested on RPE production, including WNT antagonists (Dkk-1), bone morphogenetic protein (BMP) antagonist (noggin), activin A, antagonists of NODAL (e.g., lefty-A, a transforming growth factor-beta (TGF-β) ligand), insulin-like growth factor (IGF), and small chemical molecules, such as nicotinamide (vitamin B3), dorsomorphin, XAV939, SB431542, and heparin [45]. The timescales for the production of RPE cells differ between those reports [46]. In Fig. 2, we have summarized a selected protocol for the direct differentiation of hiPSCs into RPE cells [11, 12, 47–51].

Based on the investigation of developmental studies, scientists have designed different protocols with different combinations of cytokines and small chemical molecules [52]. For instance*,* Leach et al*.* published a reliable and rapid protocol for direct differentiation, in comparison with spontaneous differentiation protocols, that allow the efficient differentiation of RPE from iPSCs by combining factors such as activin A and nicotinamide (NIC) [53], in addition to noggin, FGF-2, IGF-1, Dkk1, CHIR99021, N2, and B27 supplements that improved the efficiency of hiPSCs-derived RPE cells [53, 54]. Another optimized timing protocol leads to 60% differentiation of iPSCs into RPE within 14 days, characterized by addition of noggin, Dkk1, IGF-1, nicotinamide, or aminobenzamide at a specific time into the iPSC culture medium before activin A and VIP are added [13].

There is still controversy surrounding the immunogenicity of iPSCs and their derivatives, despite a report that differentiated cells from iPSCs are unlikely to be rejected by the immune system because they exhibit a limited immune response [55]. Additionally, even though iPSCs have been used in clinical trials using autologous cells for the first time, the high cost of cell production significantly limits their application to standard treatment. To resolve these issues, Takahashi et al. studied allogeneic retinal cell lines derived from iPSCs. In view of the fact that MHC molecules on RPE cells, including iPSCs, may be the main antigen in allogeneic inflammatory reactions, they established completely safe retinal pigment epithelial (RPE) cells from induced pluripotent stem cells (iPSCs) in homozygote major histocompatibility complex (MHC) donor animals for the transplantation. For direct differentiation of iPSCs into RPE cells, they used chemicals (signal inhibitors SB431542, Y-27632, and CKI-7) in the culture medium. After that, they transplanted allogenic iPSC-derived RPE cells into the subretinal tissue of an MHC-controlled monkey animal model. On the basis of immunohistochemical data, In MHC-matched animal models without immunosuppression, the researchers observed no rejection signs in iPSC-derived RPE allografts, but in MHC-mismatched animals, they observed immune attacks around the graft and retinal tissue damage [56].

Sugita et al. (2020) developed an optimized good manufacturing practice (GMP)-compliant protocol for the conversion of iPSCs into RPE cells, in which human iPSCs were cultured on dishes coated with gelatin. In their protocol, signal inhibitors Y-27632, CKI-7, and SB43542 were added to the GMEM medium along with a knockout serum replacement. Following the observation of RPE-like colonies, they changed the medium to DMEM supplemented with B27 and L-glutamine. They also added SB431542 and FGF-2 to the culture [50].

Zhu et al. utilized sequential retinal inducer factors (e.g., noggin, Dkk1, IGF-1, and FGF-2) and RPE specification factors (e.g., activin A, nicotinamide, and VIP) to generate RPE from hiPSCs [41]. Yang et al. examined the effects of implanting hiPSCs-derived RPE on retinal regeneration in Pde6b knockout rats in terms of retinal degeneration. hiPSCs were treated with chemicals (nicotinamide, noggin, Dkk-1, IGF-1, activin-A, SU5402, CHIR99021, Fasudil, and ROCK inhibitor) serially for 14 days in order to differentiate toward RPE cells. Afterward, the researchers injected newly generated RPE cells into rat’s eye subretinal space before evidence of retinal degeneration appeared. A significant number of transplanted cells persisted for the first 4 months; however, they gradually declined; after 10 months of transplant, they tested the cells using fundus photography, optical coherence tomography, and histology and found no evidence of abnormal cell proliferation [51]. As newly generated RPE cells have naive morphology and RPE-specific markers, one study used several cell surface markers, including CD140b, CD56, CD104, CD184, and GD2, to evaluate the maturation and purity of hiPSC-RPE differentiation. These markers may help isolate and quantify of RPE cells during differentiation in vitro, as well as improve differentiation efficiency [57]. In order to determine RPE generation, histological assessment, gene expression analysis, immunofluorescence, and FACS analysis are typically used. In addition, transepithelial resistance (TER) and phagocytosis assays are used to evaluate RPE cell function. Co-culturing of hiPSC-derived RPE cells with mouse retinal explants or RGCs (retinal ganglion cells) introduces a strategy that may lay the foundation for upcoming clinical cell therapy approaches to treat degenerated retina. In that strategy, a trans-well insert was used to separate hiPSC-RPE cells from retinal explants. According to the TUNEL staining results, when hiPSC-RPE was co-cultured with retinal explants, apoptosis was significantly lower than in the non-co-cultured control group after 2 days; RGC cultures without co-culturing hiPSC-RPE cells were used as controls. In those study, the viability and functional properties of the hiPSC-derived RPE cell were improved by the mentioned 3D culture. According to this study, transplanted hiPSC-derived RPE cells survived in the retinas of rd10 transgenic mice seventy percent of the time after implantation when stained with human nuclear antigen. The transplanted area showed a significant increase in pigment epithelium-derived factors. The transplantation of hiPSC-RPE cells also improved light avoidance behavior and ERG visual function in rd10 mice. CD68 and microglia activation markers also decreased in expression after transplantation [12]. Although many protocols for RPE differentiation require 3D structure formation, it is important to mention that the 3D method generally produces low yields of RPE [41, 58].

In a recent study, Michelet et al. introduced a simplified 2D culture in combination with lipoprotein uptake-based sorting (called the PLUS protocol) to derive an RPE monolayer from hiPSCs within 90 days. The author mentioned that differentiation of RPE by this protocol obviates the need for growth factors and small chemical molecules; thus, the production of RPE by this protocol is more cost-effective. A feeder-free culture system is also preferred [59].

For clinical uses of hiPSCs, Takao Kuroda et al. preferred a feeder-free culture in their reports [60]. They showed efficient differentiation of “primed” to “naïve” state hiPSCs toward RPE by transient inhibition of the FGF/MAPK signaling pathway. This inhibition resulted in the differentiation of neural cells and subsequent RPE generation. They also showed that BMP or PKC pathway inhibition could efficiently elevate the production of the RPE phenotype when those inhibitions are combined with FGF/MAPK inhibitors [60]. Zahabi et al. described a short-term and simple protocol to generate RPE from hiPSCs by serial addition of small chemicals (e.g., noggin, FGF-2, sonic hedgehog (Shh), and retinoic acid) in a serum-free and feeder-free adherent condition [46]. Other efforts to induce RPE from iPSCs pointed out that an animal or plant-derived biomimetic scaffold can provide favorable conditions that simulate the maturation of an RPE sheet and its integration as a functional tissue for subsequent clinical applications. Due to sterility and pro-inflammatory challenges associated with animal-derived scaffolds, researchers in one study used nanofibrous scaffolds generated from natural proteins. In order to differentiate iPSCs into RPEs, cells were cultured in a neural induction media for 22 weeks, and then in a retinal differentiation media supplemented with B27, vitamin A, ROCK inhibitor, and Y-27632 along with other essential culture medium components until achieving an epithelial-like hexagonal morphology and tight cellular packing under light microscopy [61].

In another model of differentiation, Ye et al. found that sequential treatment with inhibitors of signaling pathways (LDN193189, A-83-01, IWR-1-endo, and Y-27632 for the first 6 days followed by CHIR99021 and SU5402 for another 12 days) plus nicotinamide can increase the purity and quality of RPE sheet generation. In their experiments, they did not use any artificial scaffolds for RPE sheet transplantation since artificial scaffolds may cause inflammation; also, they did not report any tumor formation and immune rejection after transplantation. As mentioned in the Ye et al. study, effectual production of pure RPE sheets combined with the assistance of a noninvasive model that used F-actin-labeled images for machine learning-based TER prediction will be valuable for quality control and large-scale manufacturing of RPE sheet for clinic, industry, and facilitation of cell therapies [11].

In reprogramming and differentiation (e.g., trans-differentiation) studies, robust protocols have been developed to drive iPSCs differentiation to specific types of cells through overexpression of specific cell lineages transcription factors [62]. Inspired by those methods, in a recent study, it was shown that three eye-field transcription factors, *OTX2, PAX6*, and *MITF* could drive RPE differentiation in iPSCs. These transcription factors are critical regulators during eye development, the process by which anterior neuroectoderm cells become progressively specified to the RPE lineage. Overall, these transcription factors work together to promote RPE development by specifying and maintaining the eye field, the optic vesicle, and RPE [63].

According to selected references, Table 1 lists the most common signaling molecules used to differentiate iPSCs into RPEs with their roles in the signaling pathway and Table 2 summarizes the methods for differentiation of human-induced pluripotent stem cells (hiPSC) to RPE.

**Table 1:** Most frequent chemical molecules used for iPSCs differentiation toward RPEs

Chemical molecule	Role	References
Dkk-1 or XAV939	WNT signaling inhibitor, inhibits TNKS1 and TNKS2 Initiator of lens development	[12, 45, 46]
Noggin or dorsomorphin	Bone morphogenetic protein (BMP) inhibitor, AMPK pathway inhibitor, that inhibit ALK2, ALK3, and, ALK6 Induced neural fate during embryonic development from ectoderm	[13, 45, 46, 53]
Lefty-A	Transforming growth factor beta (TGF-b) ligand	[13, 45, 46, 53]
Insulin growth factor-1 (IGF-1)	Activate IGF-1 signaling receptor Stimulate increased phosphorylation in the MAPK/ERK and PI3K/AKT signaling pathways. Regulate proliferation and differentiation of RPCs	[13, 45, 46, 53]
Activin A	Expressed in neural retina, RPE during development by expression of MITF	[13, 45, 46, 53]
Nicotinamide (vitamin B3)	Inhibitor of poly-ADP ribose polymerase (PARP)	[13, 53, 64]
SB431542	Inhibitor of the TGF-β/Activin/NODAL pathway	[64, 65]
Heparin	Modulate WNT and Shh signaling pathways	[46]
CHIR99021	GSK3β inhibitor	[53, 54]
VIP (vasoactive intestinal peptide)	Activating pp60(c-SRC) and increasing intracellular cAMP	[13]
Y-27632	Inhibitor of Rho-associated protein kinase (ROCK) signaling pathways	[11]
CKI-7	Inhibitor of casein kinase 1 (CK1)	[56]
Retinoic acid (Vitamin A)	Regulate activities of nonsteroid hormone receptors such as RARα/β/γ and RXRα/β/γ in neuroretina, RPE, periocular mesenchyme, lens, cornea, iris/ciliary body, choroid, sclera, and conjunctiva	[45]
Sonic hedgehog (Shh)	Cause cell growth, cell specialization, and normal shaping; it also activates VAX1, VAX2, and PAX2 to establish both proximal–distal and dorsal–ventral axes	[46]
LDN193189	Inhibitor of BMP pathway by inhibition of ALK1, ALK2, ALK3, and ALK6	[11]
A-83-01	Inhibitor of TGFβ kinase/activin receptor-like kinase (ALK 5)	[11]
IWR-1-endo	Inhibitor of WNT pathway; AXIN2 stabilizer	[11]
SU5402	Inhibitor of MEK/ERK pathway, VEGFR2, FGFR1, and PDGFRB	[11]

**Table 2:** Summary of methods for differentiation of human-induced pluripotent stem cells (hiPSC) to RPE

References	Type of cells	Cell product	Differentiation methods and duration of follow-up	Procedure validation methods	Cell delivery method	Animal models	Assay following transplant	Research outcome (study conclusion)	Problem
[11]	hiPSC	RPE	Direct differentiation; LDN193189, A-83–01, IWR-1-endo, Y-27632, CHIR99021, SU5402 34–36-day follow-up	• Real-time PCR • Immunocytochemistry • Transepithelial electrical resistance • Phagocytosis assay • F-actin labeled imaging	RPE sheet	NA	• NA	• Obtaining high-purity RPE cells and mature RPE sheets without special selection • An automated, noninvasive TER prediction model based on F-actin-labeled images is developed to identify RPE sheets with low TER	• Sample size limitation for model training • The limited reliability of prediction models; because each manufacturer must demonstrate its own manufacturing process, safety, and efficacy of its cellular products
[12]	hiPSC	RPE	Direct differentiation; Noggin, IGF1, nicotinamide, Dkk1, bFGF, activin A, SU5402, VIP 24-day follow-up	• Immunocytochemistry • Co-culture system • Flow cytometry • TUNEL assay • 3D spheroid culture to culture spheroid RPE cells • Viability assay of spheroid RPE cells • Transepithelial electrical resistance	RPE cell suspension	Retinal degeneration 10 (rd10 mice)	• Enzyme-linked immunosorbent assay • Hematoxylin and eosin staining • Western blots • Light avoidance behavior testing • Electroretinography	• No evidence of rejection or tumorigenesis after subretinal injection for at least 2 weeks after transplantation • Co-culturing RPE by retinal explant or RGC confirmed the neuroprotective effect of secreted growth factors for retinal cells and retinal homeostasis	• 2 weeks is a rather short observation period postoperatively
[42]	hiPSCs	RPE	Spontaneous differentiation; Removal of bFGF from the medium 60–90 days up to 8 months for different cell line follow-up	• Morphological assessment • Quantitative real-time PCR • Immunocytochemistry • Immunoblot analysis • ROS phagocytosis	NA	NA	• NA	• Increases RPE65 protein expression	• Different iPSC lines exhibit different propensities to spontaneously produce RPE
[46]	hiPSCs and retinal disease-specific hiPSCs	RPE sheet	Direct differentiation; Noggin, bFGF, retinoic acid, and Shh 40-day follow-up	• Flow cytometry • Immunofluorescence • Real-time PCR	NA	NA	• NA	• Differentiation of retinal disease-specific hiPSCs toward RPE; however, it was lower in comparison with normal hiPSCs	• Additional assays such as quantitative ROS, phagocytosis, transepithelial resistance measurements, enzyme-linked immunosorbent and retinoid metabolism is also necessary for additional characterization of RPE
[47]	hESC or hiPSC	RPE	Direct differentiation; Y-27632 ROCK inhibitor, LDN/SB, SB-431542, BMP 4/7, activin A based on adherent monolayer Culture using xeno-free conditions 45-day follow-up	• Real-time PCR • Enzyme-linked immunosorbent assay • Bead phagocytosis assay • Microarray Analysis • Immunocytochemistry	NA	NA	• NA	• Efficiently direct differentiation of pluripotent stem cells toward retinal pigment epithelium fate by using a simple culture Stepwise modulation of activin A and BMP signaling method	• Further work comparing the function of RPE derived from spontaneous and directed differentiation in an in vivo setting is needed
[48]	hiPSC	RPE	Direct differentiation; Chetomin with nicotinamide 30-day follow-up	• Real-time PCR • Flow cytometry • Immunostaining • Photoreceptor outer segment phagocytosis • VEGF and PEDF ELISA	Cell suspension	NOD-SCID mice	• Fundus photographs • Immunostaining (2 weeks post-transplantation)	• A high-throughput quantitative PCR screen was combined with a new RPE reporter assay based on hiPSCs to strongly induce the conversion of over half of the differentiating cells into RPE • A single passage of the whole culture produced a highly pure RPE cell population with many of the morphological, molecular, and functional characteristics of native RPE	• There were no pigmented colonies when cultures were maintained in default medium during chetomin or chetomin/nicotinamide, suggesting that CTM-committed RPE cells are not fully mature in DM and require RPEM in order to attain their characteristic morphology
[49]	Oncogene mutation-free clinical-grade AMD patients-hiPSC	Clinical-grade RPE patches on PLGA scaffolds	Direct differentiation; LDN-193189, SB431452, CKI-7 hydrochloride, IGF-1, PD0325901, nicotinamide, activin A 6-week follow-up for cell suspension and 10-week follow-up for patch RPE	• Real-time PCR • Trans-epithelial resistance • Hexagonality measurement • Phagocytosis of photoreceptor outer segments • Lactic acid measurements	Cell suspension or patch (on PLGA Scaffold)	RCS rat Pigs with laser-induced RPE injury	• Optokinetic tracking • Optical coherence tomography and fluorescein angiography • Multi-focal Electroretinography • Immunostaining	• A biodegradable PLGA scaffold approach improved integration and functionality of clinical-grade RPE patch in rats and porcines	• No complete dose–response study was performed • An extensive set of reagents and quality control measures are required to ensure process consistency and reproducibility, which may increase the cost of manufacturing autologous cell therapy on a commercial scale
[51]	hiPSC	Retinal cells	Direct differentiation; Noggin, Dkk-1, IGF-1, nicotinamide, FGF2, activin A, SU5402, CHIR99021, ROCK inhibitor 14-day follow-up	• Morphology • Immunocytochemistry	Cell suspension	Pde6b knockout rats and SD rats	• 10-month follow-up • OCT imaging • ERG recording • Conventional PCR for validation of human mitochondrial DNA and Sanger sequencing • Hematoxylin and eosin staining • Immunohistochemical	• Transplanted human iPSC-derived retinal cells exhibited characteristics of both RPE cells and photoreceptors • No abnormal cell proliferation nor morphological changes were observed in the subretinal space	• After transplantation, the number of cells gradually decreased • The study did not reveal the presence of a network of retinal nerve cells in the region of transplantation or the linear stratification of mature retinal cells
[57]	hiPSC or hESC	RPE	Direct Differentiation; ROCK inhibitor (Y-27632), activin A More than 5-week follow-up	• Flow cytometry • Immunofluorescence • Hematoxylin–eosin staining • Immunostaining • Phagocytosis assay • Enzyme-linked immunosorbent assay • Transepithelial electrical resistance measurements • Scanning electron microscopy • Transmission electron microscopy • Single-cell RNA-sequencing bioinformatic analysis	Cell suspension	White albino rabbits	• Bright-field imaging • Immunofluorescence	• Identifying cell surface markers for RPE cells that can be used to develop a robust, direct, and scalable monolayer differentiation protocol as well as RPE cells isolation during in vitro differentiation with high quality and efficiency	• An extensive analysis would be useful to determine whether the presence of eye-field progenitors in cell suspension would be beneficial or detrimental to functional integration following subretinal transplantation • The function of RPE cells in the retina must be confirmed after the transplantation of RPE cells
[59]	hiPSC	RPE monolayer	Activin A + simplified 2D culture in combination with lipoprotein uptake-based sorting (called the PLUS protocol) 90-day follow-up	• Immunocytochemistry on cyst cryosections • Phagocytosis assay • AcLDL uptake assay • Real-time PCR analyses • Electron microscopy analyses • Enzyme-linked immunosorbent assay (ELISA) for vascular endothelial growth factor (VEGF) • Transepithelial electrical resistance measurements • Fluorescence-activated cell sorting of Dil-AcLDL positive cells	NA	NA	• NA	• This protocol obviates the need for growth factors and small chemical molecules and also is cost-effective	• To ensure the safety of these RPE cells in clinical settings, it is vital to consider the implications of transplanting trace amounts of DiI-AcLDL as well as the safety of carbocyanine dyes alone or in conjunction with lipoproteins
[61]	hiPSC	RPE cell sheet on Soy Scaffold	Direct differentiation; ROCK inhibitor (Y-27632) 5–25-week follow-up	• Immunohistochemistry • Enzyme-linked immunosorbent assay • RNA-seq analysis	NA	NA	• NA	• By cultivating RPE differentiated from hiPSCs on nanofibrous biomaterial scaffolds, whether synthetic or natural, a uniform expression of RPE maturation genes can be achieved • To evaluate the quality of differentiation on various substrates, RNA sequencing was applied	• There is a need for a variety of assays, including structural, molecular, and physiological characteristics
[63]	hiPSC and best disease patient-iPSCs	RPE monolayer	Direct differentiation; OTX2, PAX6, and MITF transcription factors + Y27632, LDN-193189, SB-431542 At least 60-day follow-up	• Real-time PCR • Immunocytochemistry • Flow cytometry • Phagocytosis with bovine rod outer segments • Western blotting • Bulk RNA-sequencing • RNA-seq data processing and analysis of differentially expressed genes • Automated 96 well plate imaging and analysis	NA	NA	• NA	• A high-efficiency and easily scalable differentiation strategy for generating iPSC-RPE from multiple patients and two wild-type iPSC lines by inducing the expression of OTX2, PAX6 and MITF (hOPM) by doxycycline paired with a small molecule • It is more appropriate to optimize differentiation requirements based on the cell line rather than the mutation in the disease	• Aside from RPE differentiation induced by specialized media, neural retina neurons are also produced. In this case, it was necessary to express hOPM in order to drive the majority of iPSCs into the RPE lineage which could result in tumorigenesis or mutation; therefore, there is a need to monitor for either of these factors
[66]	HiPSC and patient’s specific iPSC	RPE	Spontaneous differentiation; xeno-free XVIVO-10 medium without basic fibroblast growth factor 90-day follow-up Direct differentiation; nicotinamide, noggin, Dkk1, IGF1, activin A, SU5402, and CHIR99021 14-day follow-up	• Nicotinamide, noggin, Dkk1, IGF1, activin A, SU5402, and CHIR99021 • Real-time quantitative polymerase chain reaction • Next-generation sequencing (RNA-seq) • Immunocytochemistry • Rod outer segment phagocytosis assay • Pigment epithelium-derived factor enzyme-linked immunosorbent assay	NA	NA	• NA	• Directed differentiation is a more reliable method for differentiating RPE from various pluripotent sources, and some iPSC lines are more capable of RPE differentiation. Extended culture times are needed for a fully mature RPE	• It may be necessary to use directed methods rather than longer, spontaneous methods in some iPSC lines in order to [1] produce enough cells for characterization and [2] silence residual somatic cell lineage makers, both of which may require directed approaches over longer, spontaneous methods
[60]	hiPSC	RPE	Direct differentiation; PD0325901, PD173074, Gö6983, LDN-193189, CHIR99021, SB431542, SAG, SU5402, CKI-7 and Fasudil was substituted for Y-27632 30-day follow-up	• RT-PCR analysis • Immunofluorescence • Purity assay • Enzyme-linked immunosorbent assay (ELISA) for pigment epithelium-derived factor (PEDF) • Transepithelial electrical resistance measurement Phagocytosis assay	NA	NA	• NA	• During the hiPSC maintenance period, transient inhibition of the FGF/MAPK pathway promotes functional RPE differentiation and eliminates the need for subsequent treatment with WNT and nodal signal inhibitors Further inhibition of PKC or BMP signal increased differentiation efficiency	• To reduce safety risks, such as product contamination, and to reduce manufacturing costs, the number of compounds in a drug formulation should be as small as possible • To maximize yield, the culture conditions need to be optimized to maximize target cell differentiation

## Route of iPSC-derived RPE cell transplantation for AMD

There are two methods for RPE cell therapy of the retina: RPE cell suspension injection and the insertion of a monolayer patch of RPE sheet seeded on special scaffolds into the subretinal space (1). A simple and well-tolerated method in comparison with other transplantation methods is pars plana vitrectomy followed by a small incision in the damaged area in the temporal part of the macula to inject viable RPE cell suspension (around 50,000 cells per injection) into the subretinal space [12, 13]. This delivery system has several drawbacks, i.e., the risk that RPE cells flow into the vitreous cavity and PVR (proliferative vitreoretinopathy) formation and cell damage due to stress that began by cells released through the cannula [11]. Other concerns, based on available evidence, are less viable RPE cells that are unable to form an RPE monolayer as compared to a monolayer patch of RPE cells [67]. RPE cells need to form a monolayer and tight junction with adjacent RPE cells to be stably effective in the eye. They also should be fully polarized and interact with the photoreceptors to efficiently play their physiological functions [68]. In the “RPE Patch” technique, monolayer RPE cell sheets alone or laying on a biocompatible scaffold are implanted under the retina [24]. In this injection system, an oriented, polarized, and matured RPE monolayer sheet can repair the damaged area of Brunch’s membrane. A biocompatible scaffold mimics the Brunch’s membrane properties, such as permeability to soluble substances from the choroidal vessels, and supports the RPE metabolism, adhesion, and polarity. It is reported that using the scaffolds for the RPE patch method provides cellular viability and stability. It also regulates the differentiation of RPE cells in the patch and provides an efficient cellular function for them [24]. In the patch system injection, the RPE cells flow from the graft is limited. So, the main objective of this delivery system is to replace the damaged RPE layer and the Bruch’s membrane by accelerating graft integration inside the diseased microenvironment [13].

To date, the only hiPSC-derived RPE transplantation study was conducted on the implantation of scaffold-free RPE sheets to treat chronic wet AMD [50]. In that study, they used a patch strategy for autologous transplantation of iPSCs-derived RPE grew on a type I collagen scaffold in a clinical trial [46, 50]. In this model, the collagen is enzymatically dissolved, resulting in a monolayer sheet of RPE that is free from a basement substrate for surgery [46]. An iPSC-derived RPE patch with no additional scaffold was examined in one patient with non-treatable wet AMD; the vision of this patient improved and remained stable. However, it is not possible to assess the effectiveness of implanted RPE sheet in the long term with just one patient [69].

The safety and the feasibility of RPE cell suspension injection and RPE patch implantation approaches were checked in phase I/II clinical trials and have shown promising results [13]. For the clinical study, the optical coherence technology (OCT) technique was used for localization of the injection area with an incomplete loss of RPE and photoreceptors to enhance the integration possibility. For all transplantation methods, patients were immunosuppressed before and several days after the surgery [13]. Other reported approaches for delivery of stem cells in retinal diseases include intravenous administration, intravitreous (IVT) injections, and supra-choroid space injections [70].

## Safety and efficiency concerns for clinical-grade cell transplantation

According to several studies, the morphology and function of iPSC-derived RPE cells were similar to naïve RPE in vivo and in vitro [71]*.* Despite progress in stem cell research, scientists are faced with different challenges such as ethical issues, regulatory controversies, safety, and efficacy, along with the technical difficulties of adjusting this method into a standard approach for clinical application. Before iPSCs can be considered a reliable cell source for clinical-grade purposes, a variety of concerns should be taken into consideration [71]. For instance, transplanting autologous adipose stem cells, with minimal evidence of safety or efficacy, into the eye of three patients with severe AMD caused vitreous hemorrhage, PVR formation, ocular hypertension, retinal detachment through neovascularization, and lens displacement [71].

iPSC-RPE transplantation is the most challenging procedure due to immune responses. Clinical trials have noted that RPE allografts failed to survive due to immune rejection [72]. The rejection of cells after transplantation can be attributed to the degree of differences between the histocompatibility of the donor and the recipient, and published data emphasize consideration of autologous donor cells or immunologically matched cells for transplantation of RPE cells to eliminate chronic immune responses [73]. Thus, in a recent study, researchers used allogeneic RPE cells derived from the HLA-homozygous iPSC bank [74].

Making the target cells free of pluripotent stem cells is also a major challenge in cell therapy. Undifferentiated pluripotent stem cells are master cells that can create various cells of the three embryonic germ layers, and they can carry the risk of tumor formation. iPSCs were transplanted subcutaneously into immunosuppressed mice in a study to confirm their tumorigenicity. This study showed that transplanted iPSCs are tumorigenic and able to evade immune detection [75]. Hence, an extensive assay for verification of the absence of tumorigenicity and unwanted migration of the undifferentiated cells before the transplantation is necessary [32].

Generic mutations in iPSCs or their derivation are of concern since they increase the risk of cancer development in patients and the risk of transformation of the cells. For instance, the initial Japanese study that used autologous iPSC-derived RPE cells for the treatment of AMD was halted because reprogrammed iPSCs from the second patient showed unexpected mutations [71]. Therefore, genotyping and a 20-metaphase karyotypic analysis of the reprogrammed iPSCs should be performed to investigate any unwanted abnormality [76]. To reduce any risk of gene alterations, DNA-free methods, using reprogramming proteins [77] or a combination of small chemical molecules [78], have been investigated to induced pluripotency in fibroblasts. In recent studies, the use of virus-free, xeno-free, c-MYC-free, and feeder-free methods has been adopted from published studies to develop a new protocol for clinical-grade iPSC from human cells [74]. Other concerns in the field of cell therapy are the possibility of genetic mutations leading to cancer, which may occur during the in vitro derivation of iPSCs [79].

Prior to clinical-grade use of iPSC and its derivations, it is necessary to check for cross-contamination of the cell lines [80].

Since stem cells cannot decontaminate themselves, their microbiological sterility is vital in order to prevent mycoplasma, bacterial, viral, and fungal contamination, which is evident in cell transplantation therapies [81]. A complete viral testing program is required for all human adventitious agents (e.g., HBV, HCV, HIV, and nucleic acid testing). As in most laboratories, iPSCs are created by reprogramming with viral factors; the remaining reprogramming vectors in the desired cells should also be checked to ensure the safety of the reprogrammed cells [82]. The viable cell count before transplantation is another important factor to consider, as well as testing its doubling time, as this provides information on genetic stability over time. It is also mandatory to immunostaining iPSCs or target cells with at least two specific markers [83].

It is crucial for clinical-grade iPSCs to have high efficacy during reprogramming. Studies demonstrate that small-molecule inhibitors (e.g., the P38 pathway, TGF-β receptor, inositol trisphosphate 3-kinase, and Aurora A kinase) can increase the efficacy of the reprogramming procedure significantly [42].

## Conclusion

A multitude of therapeutic options based on stem cells has been explored over the last several decades. Since induced pluripotent stem cells (iPSCs) are less immunogenic and have less ethical controversy than hESC-based therapies, exploring their therapeutic potential is particularly intriguing. iPSC-derived RPE transplants became available novel treatment to humans after a decade of preclinical studies to restore vision for the patient who suffers from AMD. There has been enough evidence produced so far to confirm the safety of these potential therapeutic approaches in phase I/II clinical trials. Thus, there is probably less time to go until we have a stem cell-based treatment for acute wet AMD since only RPE cells with Bruch's membrane need replacing. However, we are far from being able to treat late dry AMD because the chronic loss of RPE will also result in secondary loss of photoreceptors overlying the affected retina. As discussed in this review, further studies must take advantage of the manufacturing process and subretinal delivery of the transplanted cell to improve the efficacy of RPE fabrication and their integration into the retina as well as improve the retina microenvironment for long-term integration and survival of transplanted cells.

## Data Availability

Not applicable.

## Abbreviations

ALK: Activin receptor-like kinase (ALK)
AMD: Age-related macular degeneration
Anti-VEGF: Anti-vascular endothelial growth factor
ARMS2: Age-related maculopathy susceptibility gene 2
AXIN: Axis inhibition protein
A2E: N-retinyl-N-retinylidene ethanolamine
BMP4: Bone morphogenetic protein 4
BRB: Blood–retinal barrier
CD140b: Anti-PDGF receptor beta/PDGFR-β
CD104: Integrin beta 4
CD184: C-X-C chemokine receptor type 4
CD56: Neural cell adhesion molecule
CFH: Complement factor H
CKI-7: Casein kinase 1 (CK1) inhibitor
C-Myc: Cellular myelocytomatosis
DKK-1: Dickkopf-related protein 1
DMEM: Dulbecco's modified eagles’ medium
EGF: Epidermal growth factor
ERG: Electroretinography
ERK: Extracellular signal-regulated kinase
ESC: Embryonic stem cell
FACS: Fluorescence-activated cell sorting
FGFR: Fibroblast growth factor receptor
FGF2: Fibroblast growth factor 2
GD2: Disialoganglioside
GMEM: Glasgow minimum essential medium
GMP: Good manufacturing practice
GSK3β: Glycogen synthase kinase 3 beta
hESC: Human embryonic stem cell
hiPSC: Human-induced pluripotent stem cell
HLA: Human leukocyte antigen
hPSC: Human pluripotent stem cell
IGF-1: Insulin-like growth factor-1
iPSC: Induced pluripotent stem cell
iPSC-RPE: Induced pluripotent stem cell-derived retinal pigment epithelium
IVT: Intravitreal injection
IWR-1endo: Wnt/β-catenin signaling inhibitor
KLF4: Kruppel-like factor 4
MAPK: Mitogen-activated protein kinase
MEK: Mitogen-activated protein kinase
MITF: Microphthalmia-associated transcription factor
NIC: Nicotinamide
NODAL: Nodal growth differentiation factor
OCT: Optical coherence tomography
OCT4: Octamer-binding transcription factor 4
OTX2: Orthodenticle homeobox 2
PAX2: Paired box protein Pax-2
PAX6: Paired box protein Pax-6
PDGFRβ: Platelet-derived growth factor receptor beta
PKC: Protein kinase C
PVR: Proliferative vitreoretinopathy
RPCs: Retinal progenitor cells
RPE: Retinal pigment epithelium
RGCs: Retinal ganglion cells
ROCK inhibitor: Rho-associated protein kinase inhibitor
Shh: Sonic hedgehog
SOX2: SRY-box 2
SRC: Proto-oncogene tyrosine-protein kinase Src
TIMP3: TIMP metallopeptidase inhibitor 3
TNF: Tumor necrosis factor
TNKS: Tankyrase
TUNEL: Terminal deoxynucleotidyl transferase dUTP nick end labeling
TER: Transepithelial resistance
TGF-β: Transforming growth factor-beta
VAX: Ventral anterior homeobox
VEGF: Vascular endothelial growth factor
VIP: Vasoactive intestinal peptide
WNT: Wnt is a portmanteau created from the names Wingless and Int-1
